# The use of the SPT-based seismic soil liquefaction triggering evaluation methodology in engineering hazard assessments

**DOI:** 10.1016/j.mex.2018.11.016

**Published:** 2018-11-27

**Authors:** K. Onder Cetin, Raymond B. Seed, Robert E. Kayen, Robb E.S. Moss, H. Tolga Bilge, Makbule Ilgac, Khaled Chowdhury

**Affiliations:** a Dept. of Civil Engineering, Middle East Technical University, Ankara, Turkey; b Dept. of Civil and Environmental Engineering, University of California, Berkeley, CA, USA; c California Polytechnic State University, San Luis Obispo, CA, USA; d Civil-Geotechnical Engineer, GeoDestek Ltd. Sti., Ankara, Turkey; e US Army Corps of Engineers, South Pacific Division Dam Safety Production Center, Sacramento, CA, USA

**Keywords:** A simplified probabilistic and deterministic SPT-based liquefaction triggering assessment methodology, Soil liquefaction, Earthquake, Seismic hazard, Cyclic loading, Standard penetration test, In-situ test, Probability

## Abstract

Probabilistic and deterministic seismic soil liquefaction triggering methodologies are proposed in Cetin et al. [1]. This manuscript: i) presents the protocols, which need to be followed for the correct use of this methodology for forward engineering (design) assessments, ii) guides the engineers through the procedure, and iii) discusses the “tricks” alongside the protocol. An illustrative soil profile shaken by a scenario earthquake is presented, through which consistent estimations of representative SPT blow-counts along with fines content are discussed. Additionally, the estimation of CSR input parameters are illustrated. Last but not least the uncertainty estimations of these input parameters are presented along with the probability and factory of safety for the assessment of liquefaction triggering.

• A simplified methodology and its use to assess liquefaction triggering hazard of a soil site under an earthquake scenario event.
• The consistent and unbiased mean estimates of input parameters of SPT blow-counts($N_{1,60}$), fines content (FC), vertical effective (${\sigma '}_{v}$) and total ($\sigma_{v}$) stresses, maximum ground acceleration ($a_{max}$), stress reduction (or non-linear shear mass participation) factor ($r_{d}$) and moment magnitude ($M_{w}$) along with their uncertainties are discussed.
• Outlined methodology enables engineers to estimate the probability of- and factor of safety against- seismic soil liquefaction triggering for design problems.

## Graphical abstract

**Figure:**
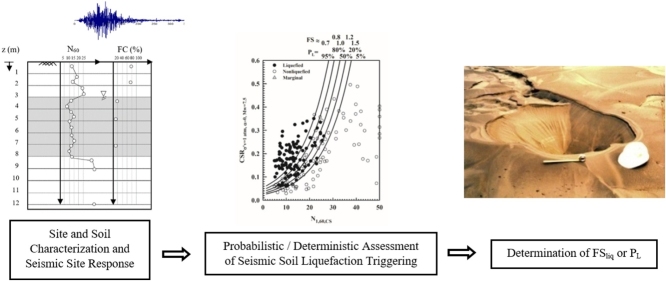

Specifications Table

**Table:** 

Subject area	• *Earth and Planetary Sciences* • *Engineering*
More specific subject area	*Civil Engineering, Geotechnical Engineering, Earthquake Engineering*
Method name	*A simplified probabilistic and deterministic SPT-based liquefaction triggering assessment methodology.*
Name and reference of original method	*Cetin et al.* [1] *Seed and Idriss* [2] *Simplified Procedure* *Cetin* [3] *Cetin et al.* [4]
Resource availability	*Cetin et al.* [1] *Cetin et al.* [5]

## Method details

### A summary of proposed SPT-based probabilistic and deterministic liquefaction triggering methodology: background

A new set of probabilistic and deterministic seismic soil liquefaction triggering relationships is presented in Cetin et al. [1], on the back analyses of standard penetration test liquefaction triggering case histories, which are fully documented in Cetin et al. [6]. The use in forward (design) assessments of these new relationships requires i) the correct understanding of the protocols behind case history processing, and ii) the consistent use of these protocols in design assessments. This manuscript is intended to discuss these protocols, and to guide engineers through the correct and consistent use of them, along with a discussion on “tricks” alongside these protocol. For this purpose, an illustrative soil profile shaken by a scenario earthquake is used to outline the use of the proposed methodology. These new relationships are given in Eqs. (1) and (2).

(1) PLN1,60,CSRσ'v,α=0,Mw,Mw,σ'v,FC=Φ-N1,60∙1+θ1⋅FC-θ6⋅lnCSRσ'v,α=0,Mw-θ2⋅lnMw-θ3⋅lnσ'vPa+θ4⋅FC+θ5σε

(2) CRRN1,60,Mw,σ'v,FC,PL=expN1,60∙1+θ1⋅FC-θ2⋅lnMw-θ3⋅lnσ'vPa+θ4⋅FC+θ5+σε⋅Φ-1(PL)θ6

**Fig. 1:**
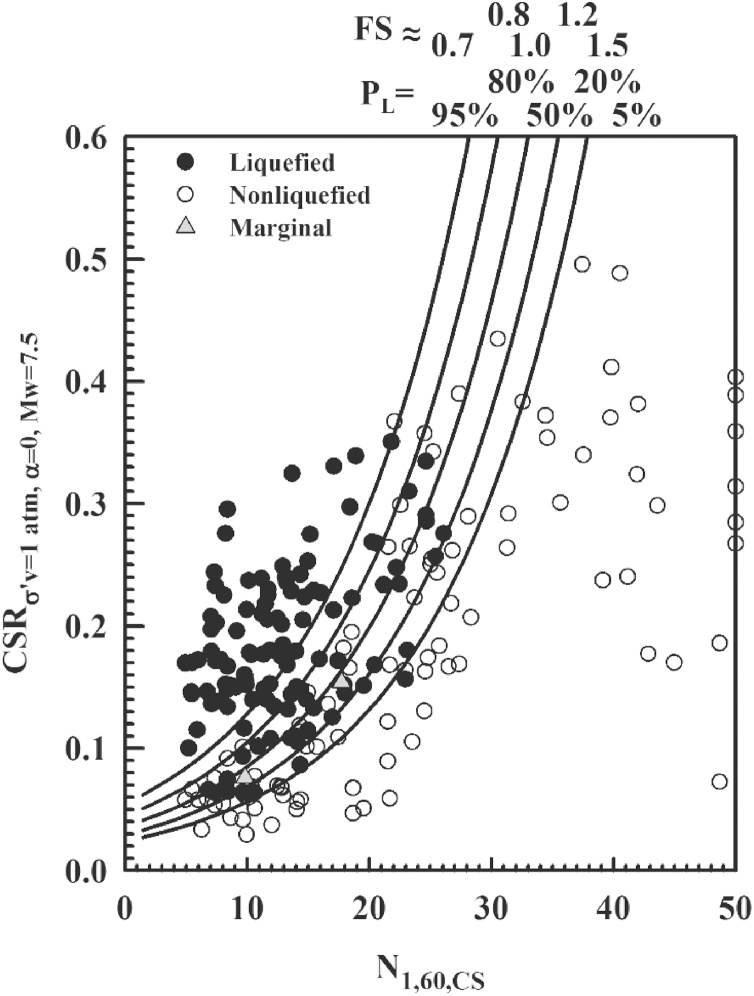
New probabilistic seismic soil liquefaction triggering curves.

In Eq. (1), $P_{L}$ is the probability of liquefaction in decimals (i.e. $P_{L}$ = 30% is input as 0.30), ${CSR}_{{\sigma^{'}}_{v},\alpha =0,M_{w}}$ is not “adjusted” for vertical effective stress or magnitude/duration effects (corrections are executed within Eq. (1) itself), FC is percent fines content (by dry weight) expressed as an integer (e.g.: 12% fines is input as FC = 12) with the limit of 5 ≤ FC ≤ 35, $P_{a}$ is atmospheric pressure (1 atm. = 101.3 kPa = 2116.2 psf) in the same units as the in-situ vertical effective stress (${\sigma^{'}}_{v}$), and Φ is the standard cumulative normal distribution. The cyclic resistance ratio (CRR), for a given probability of liquefaction can be expressed as given in Eq. (2), where ${\text{Φ}}^{-1}\left(P_{L}\right)$ is the inverse of the standard cumulative normal distribution (i.e. mean = 0, and standard deviation = 1). For spreadsheet construction purposes, the command in Microsoft Excel for this specific function is “NORMINV(P_L_,0,1)”. In Fig. 1, factor of safety (FS) values corresponding to probabilities of liquefaction 5, 20, 50, 80, 95% are also presented.

The new probabilistic boundary curves, as shown in Fig. 1, are estimated by considering the uncertainty due to model error only. In this figure, the dots and circles represent liquefied and non-liquefied case histories compiled from available literature. As stated earlier, a complete documentation of these case histories including their source references is presented in Cetin et al. [6]; hence will not be repeated herein. These new triggering relationships, which are discussed in detail in Cetin et al. [1], will be referred to as CEA2018 (Cetin et al.), hereafter. The values of the model coefficients (i.e. $\theta_{i}$) as defined in Eqs. (1) and (2), are listed in Table 1.

**Table 1:** A summary of model coefficients of CEA2018 seismic soil liquefaction triggering relationship.

$\theta_{1}$	$\theta_{2}$	$\theta_{3}$	$\theta_{4}$	$\theta_{5}$	$\theta_{6}$	$\theta_{7}$	$\sigma_{\varepsilon }$
0.00167	27.352	3.958	0.089	16.084	11.771	0.392	2.95

Approximate factors of safety, FS values can be estimated by Eq. (3) based on the assumption that $P_{L}$ of 50% corresponds to a best-estimate factor of safety value of 1.0.

(3) $$FS=\frac{CRR(P_{L}=50\text{\%})}{CRR(P_{L})}=exp\left[\frac{-\sigma_{\varepsilon }\cdot {\text{Φ}}^{-1}\left(P_{L}\right)}{\theta_{6}}\right]=exp\left[\frac{-2.95\cdot {\text{Φ}}^{-1}\left(P_{L}\right)}{11.771}\right]=exp\left[-0.251\cdot {\text{Φ}}^{-1}\left(P_{L}\right)\right]$$

It should be noted that Eqs. (1) and (2) are applicable only if input parameters (e.g.: $N_{1,60},{CSR}_{{\sigma^{'}}_{v},\alpha =0,M_{w}},M_{w},{\sigma^{'}}_{v},FC$) are assumed to be exact (i.e. no input parameter uncertainty exists). If these parameters have uncertainties, very typical in design problems, these uncertainties can be represented by standard deviation terms of $\sigma_{N_{1,60}}$, $\sigma_{{\text{l}\text{n}(CSR}_{{\sigma '}_{v},\alpha ,M_{w}})}$, $\sigma_{M_{w}}$, $\sigma_{FC}$, $\sigma_{{\sigma '}_{v}}$, or coefficient of variation terms of $\delta_{N_{1,60}}$, $\delta_{{\text{l}\text{n}(CSR}_{{\sigma '}_{v},\alpha ,M_{w}})}$, $\delta_{M_{w}}$, $\delta_{FC}$, $\delta_{{\sigma '}_{v}}$. Then, on the basis of first order second moment reliability method, the overall cumulative variance (σ^2^_tot_) of the limit state function given in the nominator of Eq. (1) is estimated as the sum of the variance of input parameters (σ^2^_input_) and model error (σ^2^_ε_) as given in Eq. (4).

(4) $$\sigma_{tot,i}^{2}={\left(\theta_{7}\cdot \sigma_{input}\right)}^{2}+\sigma_{\varepsilon }^{2}$$

In Eq. (4), $\sigma_{input}$ represents the consolidated uncertainty of the liquefaction engineering input parameters, and as discussed in CEA2018, a scaling factor of $\theta_{7}$ needs to be systematically applied. This factor ($\theta_{7}$) is one of the regressed parameters of the overall triggering relationship. If the higher-order terms are eliminated, then $\sigma_{input}$ can be estimated as given Eq. (5).

(5) $$\sigma_{input}^{2}={\theta_{6}}^{2}\cdot {\left[\delta_{{CSR}_{{\sigma \text{'}}_{v},\alpha ,M_{w}}}\right]}^{2}+\sigma_{N_{1,60}}^{2}\cdot {\left(1+\theta_{1}\cdot FC\right)}^{2}+\sigma_{FC}^{2}\cdot {\left(\theta_{1}\cdot N_{1,60}+\theta_{4}\right)}^{2}+{\theta_{3}}^{2}\cdot {\left[\delta_{{\sigma^{\text{'}}}_{v}}\right]}^{2}$$

The estimation of the uncertainties in input parameters will be discussed later in this manuscript.

### Recommended use of the proposed liquefaction triggering assessment methodology

**Fig. 2:**
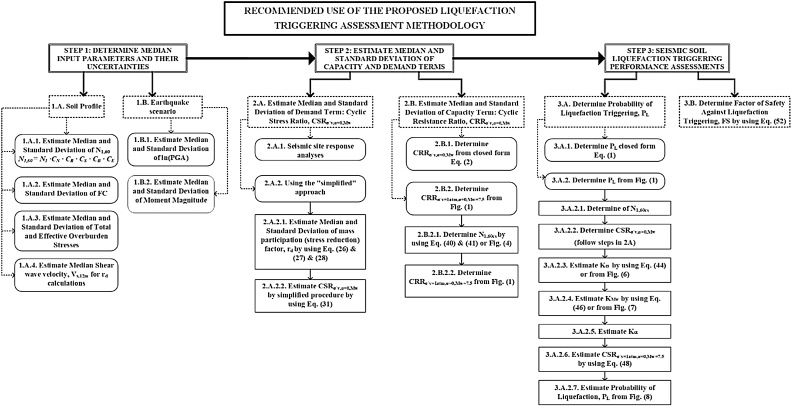
Recommended Flowchart for Liquefaction Triggering Assessments.

The proposed new probabilistic correlations can be used in two ways. They can be used directly, all at once, as summarized in Eqs. (1) and (2), oralternatively, they can be used “in parts” as most of the previous similar conventional methods. A flow chart is presented in Fig. 2, which summarizes the required assessment protocols for the consistent use of recommended methodology.

**Fig. 3:**
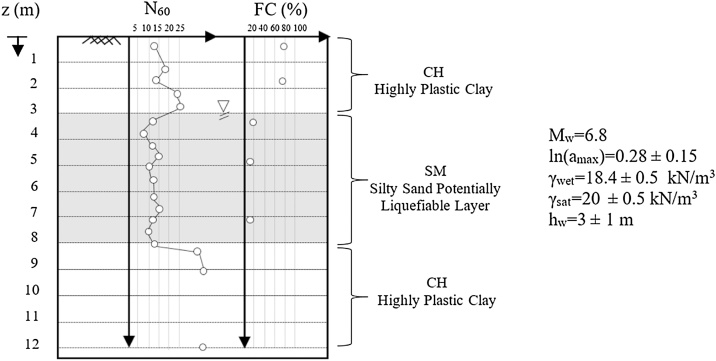
Soil profile and input parameters of the selected case for illustration of proposed methodology.

For illustrating the use of the proposed methodology and details of assessment steps described in Fig. 2, a generic site is to be assessed. As presented in Fig. 3, this illustrative soil site is composed of three layers: There exists a 3 m thick high-plasticity clay (CH) layer at the surface, which is underlain by a 5 m thick, potentially liquefiable, medium-dense silty sand (SM) layer. At and below 8 m depth, there exists another highly plastic clay (CH) layer. The earthquake scenario is deterministically selected as a M_w_ = 6.8 event, which is assumed to produce a maximum peak ground acceleration ($a_{max}$) of 0.28 g. The coefficient of variation of $a_{max}$ is assumed as 0.15. The ground water table depth ($h_{w}$) is assumed to be 3 m with standard deviation of 1 m. The other necessary input parameters are also presented along with the soil profile given in Fig. 3. Site and soil specific moist and saturated unit weights are estimated based on laboratory test results as given in the same figure.

**Step 1: Determination of Mean Input Parameters and Their Uncertainties**

The input parameters required for seismic soil liquefaction triggering assessments will be discussed under two separate sections: the ones related to a) soil profile and soil characteristics, and b) seismic scenario.

### Soil profile

#### Estimation of mean and standard deviation of N_1,60_

The critical layer has multiple and consistently scattered SPT blow counts ($N_{60}$). The field N values are corrected for effective normal stress ($C_{N}$), hammer energy ($C_{E}$), rod length ($C_{R}$), sampler ($C_{S}$), borehole diameter ($C_{B}$), and procedural effects to fully standardized $N_{1,60}$ values, as given in Eq. (6).

(6) $$N_{1,60}=N\cdot C_{N}\cdot C_{R}\cdot C_{S}\cdot C_{B}\cdot C_{E}$$

The corrections for C_N_, C_R_, C_S_, C_B_ and C_E_ correspond closely to those recommended by NCEER Working Group (NCEER [7], also given in Youd et al. [8]), and they are summarized in Table 2 for the sake of completeness.

**Table 2:** Recommended corrections for SPT equipment, energy and procedures.

C_N_	$C_{N}={\left(\frac{P_{a}}{{\sigma '}_{v}}\right)}^{0.5}\leq 2.0$ where the effective stress, ${\sigma^{\prime }}_{v}$, and reference stress,Pa, are in the same units.		
C_R_	$C_{R}=0.48+0.225\cdot \text{ln}\left(d\right)$; d ≤ 10 m (T-1) $C_{R}=0.48$; 10 m < d < 30m where d = rod length (or “stick-up”) from the top of the SPT sampler to the striking point at the top of the rod.		
C_S_	For samplers with an indented space for interior liners, but with liners omitted during sampling, $C_{S}=1+\frac{N_{1,60}}{100}$ (T-2) with limits of 1.10 ≤ C_S_ ≤1.30		
C_B_	Borehole diameter correction (C_B_) 65 to 115 mm: 1.00 150 mm: 1.05 200 mm: 1.15		
C_E_	$C_{R}=\frac{ER}{60}$ (T-3) where ER (energy efficiency ratio) is the fraction or percentage of the theoretical SPT impact hammer energy actually transmitted to the sampler, expressed as % • The best approach is to directly measure the impact energy transmitted with each blow with instrumented rod. When available, direct energy measurements were employed. • The next best approach is to use a hammer and automatic (mechanical) trip hammer release system that has been demonstrated to deliver repeatable energy, and which has been calibrated based on direct (-instrumented) energy measurements. • Otherwise, ER must be estimated. For good field procedures, equipment and monitoring, the following approximate guidelines for SPT performed with rope and cathead are suggested:		
	Equipment	Approximate ER (see Note 3)	C_E_ (see Note 3)
	-Safety Hammer^1^	0.4–0.75	0.7–1.2
	-Donut Hammer^1^	0.3–0.6	0.5–1.0
	-Donut Hammer^2^	0.7–0.85	1.1–1.4
	-Automatic-Trip Hammer (Donut or Safety Type)	0.5–0.8	0.8–1.4
	• For lesser quality fieldwork (e.g.: irregular hammer drop distance, excessive sliding friction of hammer on rods, wet or worn rope on cathead, etc.) further judgmental adjustments are needed.		

Notes: (1) Based on rope and cathead system, two turns of rope around cathead, “normal” release (not the Japanese “throw” (Seed et al. [9]), and rope not wet or excessively worn.

(2) Rope and cathead with special Japanese “throw” release. (See also Note 4.

(3) For the ranges shown, values roughly central to the mid-third of the range are more common than outlying values, but ER and C_E_ can be even more highly variable than the ranges shown if equipment and/or monitoring and procedures are not good.

(4) Common Japanese SPT practice (Seed et al. [9]) requires additional corrections for Borehole diameter and for frequency of SPT hammer blows. For “typical” Japanese practice with rope and cathead, donut hammer, and the Japanese “throw” release, the overall product of CB x CE is typically in the range of.1.0–1.3.

On the basis of Taylor’s expansion, the first-order approximations of the mean and variance of N_1,60_ are given in Eqs. (7) and (8) with the assumption that correction factors are all exact (i.e.: uncertainties in correction factors are ignored).

(7) $$\mu_{N_{1,60}≅\mu_{N}\cdot C_{N}\cdot C_{E}\cdot C_{B}\cdot C_{R}\cdot C_{S}}$$

(8) $$\delta_{N_{1,60}}≅\delta_{N}$$

For design purposes it is recommended that the field N-values within a critical layer from one or more boreholes at a soil site to be corrected as recommended in Eq. (6) to estimate $N_{1,60}$ values. These are then plotted vs. the topography-corrected depth below the ground surface. In many cases, a given soil layer will be found to contain an identifiable critical sub-stratum based on a group of localized low $N_{1,60}$ -values. Occasional high values, assumed to be gravel and not apparently representative of the general characteristics of the critical stratum, are removed if they sit outside the main cluster of points. Similarly, though less often, very low $N_{1,60}$ values that are much lower than the apparent main cluster of points representing the stratum are typically associated with locally high fines content and eliminated if the soils have significant fines and if it appears that the fines are plastic in nature. The remaining, corrected $N_{1,60}$ values are then used to evaluate both the mean of $N_{1,60}$ within the critical stratum, and the variance in $N_{1,60}$. When applicable, SPT blowcounts above the groundwater table may be also used to characterize the relative density state of the critical layer.

Table 3 presents the corrected SPT blowcounts along with the shear wave estimations, details of which will be given in Step1.A.4. After applying the corrections for the SPT blowcounts, the critical silty sand layer is characterized by the mean and the standard deviation of scattered SPT blow counts, as 11.4 blows/ft and 2.3 blows/ft, respectively. For the estimation of rod length, a 1.2 m of stick up is assumed.

**Table 3:** SPT corrections, estimation of mean and standard deviation values for $N_{1,60}$ and.FC

Depth (m)	N_60_	FC	C_N_	C_R_	N_1,60_	V_s_	CSR
0.5	13	82	2.00	0.60	15.6	235.1	–
1.2	17		2.00	0.68	23.0	257.1	
1.8	14	78	1.75	0.73	17.8	241.0	
2.2	24		1.58	0.76	28.7	288.4	
2.8	25		1.40	0.79	27.8	292.4	
***3.2***	***12***	***20***	***1.33***	***0.81***	***13.0***	***183.2***	***0.186***
***3.7***	***7***		***1.27***	***0.84***	***7.5***	***153.0***	***0.199***
***4.3***	***11***		***1.22***	***0.86***	***11.6***	***177.9***	***0.212***
***4.6***	***15***		***1.19***	***0.88***	***15.6***	***197.3***	***0.218***
***5***	***10***	***10***	***1.16***	***0.89***	***10.3***	***172.4***	***0.224***
***5.5***	***11***		***1.12***	***0.91***	***11.2***	***177.9***	***0.231***
***6.2***	***11***		***1.07***	***0.93***	***11.0***	***177.9***	***0.238***
***6.7***	***15***		***1.04***	***0.95***	***14.8***	***197.3***	***0.242***
***7.1***	***11***	***12***	***1.02***	***0.96***	***10.7***	***177.9***	***0.245***
***7.5***	***10***		***1.00***	***0.97***	***9.7***	***172.4***	***0.247***
***8.1***	***11***		***0.97***	***0.98***	***10.5***	***177.9***	***0.249***
8.3	34		0.96	0.99	32.3	324.0	0.250
9.1	35		0.93	1.00	32.5	327.1	0.251
12	35		0.83	1.00	29.1	327.1	0.243
	μ*	**14.0**		μ*	**11.4**		
	σ*	**5.3**		σ*	**2.3**		

* The values within the potentially liquefiable layer are used to estimate the mean and the standard deviation terms.

#### Estimation of mean and standard deviation of FC

The fines (silt and clay particles) of soils are widely expressed by fines content, which is defined as the portion of soil particles by mass finer than the No. 200 sieve (0.074 mm). Following the similar procedure outlined for the estimation of mean and standard deviation of $N_{1,60}$ values, the mean and standard deviation of FC values are estimated as 14 ± 5.3, respectively. Note that the FC values are limited to be within the range of 5–35 %. If any outlier data exists, it needs to be excluded or critical layer needs to be divided into more homogenous soil sub-layers. In the literature there exist different opinions regarding the effects of fines on penetration (Cubrinovski and Ishihara [10], Shahien [11]) and liquefaction resistances (Cetin et al. [1], Youd et al. [8], Tokimatsu and Yoshimi [12], Seed et al. [13], Idriss and Boulanger [14]) and on the choices of maximum fines content limits (Cetin et al. [1], Shahien and Mesri [15], Idriss and Boulanger [16]). Depending on the plasticity of fines, density state of the coarse grained portion, skeleton void ratio, soil fabric and layering, these effects may be more pronounced (Troncoso and Verdugo [17], Koester [18], Thevanayagam et al. [19], Martin and Polito [20], Huang and Zhao [21]). A more detailed discussion of these effects is presented elsewhere (Cetin et al. [1,4], and will not be repeated herein.

#### Estimation of mean and standard deviation of total and effective overburden stresses

Total and effective vertical stress estimations require the estimation of soil unit weights along with the depth of ground water table. If soil specific unit weight data is missing, the recommended values, which were also used in CEA2018 for back analyses of field performance case histories, as presented in Table 4, can be used. For this illustrative site, the mean values of moist ($\gamma_{moist})$ and saturated ($\gamma_{sat})$ unit weights are estimated based on laboratory test results as given in Fig. 3.; whereas, their standard deviations are assumed as 0.5 kN/m^3^.

**Table 4:** Assumed unit weights as used in CEA2018.

SPT-N_60_	γ_moist_		γ_sat_	
(blows/ft)	(lb/ft^3^) (kN/m^3^)		(lb/ft^3^) (kN/m^3^)	
(a) Coarse-grained soil layers				
0–4	100	15.7	110	17.3
5–10	110	17.3	120	18.9
11–30	120	18.9	125	19.6
30–50	125	19.6	135	21.2
(b) Fine-grained soil layers				
0–4	100	15.7	110	17.3
5–8	110	17.3	120	18.9
9–16	115	18.1	125	19.6

In addition to the uncertainty of the mean estimates of soil unit weights, the inexact estimation of the depth to water table affects the accuracy of vertical effective stress estimations. In the literature, clear definitions for uncertainty estimations of phreatic surface depths are not available. Based on expert opinions the following simple procedure is proposed:

1 If there are multiple borings available showing a consistent depth to water table, h_w_, and the borings are drilled at a reasonable time period before or after the earthquake (i.e. the ground water conditions have not significantly changed.), $\sigma_{h_{w}}$ ≤0.3 m (∼ 1 ft); where $\sigma_{h_{w}}$ is the standard deviation of the depth to water table.
2 For all other cases $\sigma_{h_{w}}$ >0.3 m (∼ 1 ft), on a case by case basis.

The mean values for total and effective vertical overburden stresses are estimated for the mid-depth of the critical layer as given in Eqs. (9) and (10), respectively. The uncertainty of these input parameters are estimated as given in Eqs. (11), (12), (13) on the basis of first order approximation as discussed briefly earlier in this manuscript, and in-detail in CEA2018.

(9) $$\mu_{\sigma_{v}}≅\mu_{\gamma_{1}}\cdot \mu_{h_{w}}+\mu_{\gamma_{2}}\cdot {(\mu }_{h}-\mu_{h_{w}})$$

(10) $$\mu_{{\sigma \text{'}}_{v}}≅\mu_{\gamma_{1}}\cdot \mu_{h_{w}}+(\mu_{\gamma_{2}}-\gamma_{w})\cdot {(\mu }_{h}-\mu_{h_{w}})$$

(11) $$\sigma_{\sigma_{v}}^{2}≅\mu_{h_{w}}^{2}\cdot \sigma_{\gamma_{1}}^{2}+{\left(\mu_{h}-\mu_{h_{w}}\right)}^{2}\cdot \sigma_{\gamma_{2}}^{2}+\mu_{\gamma_{2}}^{2}\cdot \sigma_{h}^{2}+{\left(\mu_{\gamma_{1}}-\mu_{\gamma_{2}}\right)}^{2}\cdot \sigma_{h_{w}}^{2}$$

(12) $$\sigma_{{\sigma \text{'}}_{v}}^{2}≅\mu_{h_{w}}^{2}\cdot \sigma_{\gamma_{1}}^{2}+{\left(\mu_{h}-\mu_{h_{w}}\right)}^{2}\cdot \sigma_{\gamma_{2}}^{2}+{\left(\mu_{\gamma_{2}}-\gamma_{w}\right)}^{2}\cdot \sigma_{h}^{2}+{\left(\mu_{\gamma_{1}}+\gamma_{w}-\mu_{\gamma_{2}}\right)}^{2}\cdot \sigma_{h_{w}}^{2}$$

(13) $$Cov\left[\sigma_{v},{\sigma \text{'}}_{v}\right]≅\left(\mu_{h_{w}}^{2}\cdot \sigma_{\gamma_{1}}^{2}\right)+\left(\mu_{\gamma_{1}}-\mu_{\gamma_{2}}\right)\cdot \left(\mu_{\gamma_{1}}+\gamma_{w}-\mu_{\gamma_{2}}\right)\cdot \sigma_{h_{w}}^{2}+{{(\mu }_{h}-\mu_{h_{w}})}^{2}\cdot \sigma_{\gamma_{2}}^{2}+\mu_{\gamma_{2}}\cdot (\mu_{\gamma_{2}}-\gamma_{w})\cdot \sigma_{h}^{2}$$

In these equations, $\gamma_{1}$ and $\gamma_{2}$ represent the moist and saturated unit weights, respectively. On the other hand, $\mu_{h}$ and σ_h_ are the mean and standard deviation of the critical depth, respectively. For the illustrative site discussed herein, $\mu_{h}$ can be estimated as 5.5 m (=((critical layer’s upper depth = 8 m) + (critical layer’s lower depth = 3 m))/2); whereas its standard deviation is estimated as 0.83 m, assuming that mean±3σ covers the complete critical depth range (i.e.: (8-3)/6 = 0.83 m). Then, by substituting the corresponding values, the mean and the standard deviation of total and effective stresses at the mid-depth of the critical layer are estimated as 105.2 ± 16.8 kPa and 80.7 ± 12kPa, respectively. The calculation steps are given in Eqs. (14), (15), (16), (17), (18), (19), (20).

(14) $$\mu_{\sigma_{v}}≅18.4\cdot 3+20\cdot \left(5.5-3\right)=105.2\;kPa$$

(15) $$\mu_{{\sigma \text{'}}_{v}}≅18.4\cdot 3+\left(20-9.81\right)\cdot \left(5.5-3\right)=80.7\;kPa$$

(16) $$\sigma_{\sigma_{v}}^{2}≅3^{2}\cdot 0.5^{2}+{\left(5.5-3\right)}^{2}\cdot 0.5^{2}+20^{2}\cdot 0.83^{2}+{\left(20-18.4\right)}^{2}\cdot 1^{2}=281.9$$

(17) $$\sigma_{\sigma_{v}}=16.8\;kPa$$

(18) $$\sigma_{{\sigma \text{'}}_{v}}^{2}≅3^{2}\cdot 0.5^{2}+{\left(5.5-3\right)}^{2}\cdot 0.5^{2}+{\left(20-9.81\right)}^{2}\cdot 0.83^{2}+{\left(18.4+9.81-20\right)}^{2}\cdot 1^{2}=142.8$$

(19) $$\sigma_{{\sigma \text{'}}_{v}}=12\;kPa$$

(20) $$Cov\left[\sigma_{v},{\sigma^{\prime }}_{v}\right]≅\left(3^{2}\cdot 0.5^{2}\right)+\left(18.4-20\right)\cdot \left(18.4+9.81-20\right)\cdot 1^{2}+{\left(5.5-3\right)}^{2}\cdot 0.5^{2}+20\cdot \left(20-9.81\right)\cdot 0.83^{2}=131.1$$

#### Estimation of mean shear wave velocity, $V_{s,12m}$ for $r_{d}$ calculations

Cetin and Seed [22] $r_{d}$ relationship requires the estimation of a representative shear wave velocity for the upper 12 m of the soil site. If in-situ Vs measurements are available they can be directly used, otherwise Eqs. (21) and (22) from the Design Specification for Highway Bridges, Japan Road Association [23] can be used as one of many N vs. V_s_ correlations. Note that the V_s_ estimations presented in Table 3 were also determined via these equations.

(21) $$V_{s}\approx 80\cdot N^{1/3}(\text{i}\text{n}\;\text{m}/\text{s})\;(\text{f}\text{o}\text{r}\;\text{s}\text{a}\text{n}\text{d})$$

(22) $$V_{s}\approx 100\cdot N^{1/3}(\text{i}\text{n}\;\text{m}/\text{s})\;(\text{f}\text{o}\text{r}\;\text{c}\text{l}\text{a}\text{y})$$

$V_{s,12m}$ is estimated by calculating the apparent travel times through each sub-layer, down to a depth of 12 m, and then by dividing the total travel time by the distance travelled, as given in Eq. (23).

(23) $$V_{s,12m}=\frac{12\;m}{\sum \frac{H_{i}}{V_{s,i}}}$$

Assuming that the average shear wave velocity values are 250, 170 and 320 m/s for the highly-plastic clay, silty sand, and claystone layers, the mean shear wave velocity for the upper 12 m is estimated as 220 m/s as shown in Eq. (24).

(24) $$V_{s,12m}=\frac{12\;m}{\left(\frac{3}{250}+\frac{5}{170}+\frac{4}{320}\right)}\approx 220\;\text{m}/\text{s}$$

### Earthquake scenario

#### Estimation of mean and standard deviation of ln(PGA)

Estimating peak ground acceleration ($a_{max}$), at soil sites requires the understanding of both the seismicity (magnitude, source mechanism, travel path, directivity effects, etc.) and the response (both geological and geotechnical) characteristics of the site. In the literature, for the assessment of liquefaction triggering case histories, peak ground acceleration has been evaluated in the order of decreasing accuracy by using:

1 Strong ground motion recordings obtained directly at the site of interest (e.g.: Wildlife site, U.S.A., and Port Island site, Japan),
2 Site response analysis tools with a good, “representative” input motion developed from an event-specific nearby ground motion record,
3 Site and earthquake specific attenuation relationships, derived from available strong ground motion data recorded on similar nearby soil sites, or on rock sites where the amplification or de-amplification of soil sites are incorporated separately, and with reasonable azimuthal accounting for directivity and travel path effects, etc.,
4 Generalized attenuation relationships (e.g.: Abrahamson and Silva [24], Idriss [25], etc.), with modifications to account for the effects of local site conditions,
5 Generalized attenuation relationships without local records to provide event specific calibration,
6 Intensity scales. (e.g.: modified Mercalli scale).

Within the scope of Cetin et al. [1] studies, case histories where $a_{max}$ cannot be estimated by one of the first three methods were eliminated from further consideration. This also defines an upper boundary on the uncertainty of the mean estimates of $a_{max}$ as to be less than that predicted by generalized attenuation relationships. In all cases, $a_{max}$ estimations adopted for these studies are based on the geometric mean of the two orthogonal components of available recordings. Typical attenuation relationships (e.g.: Abrahamson and Silva [24], Idriss [25]) can estimate peak ground acceleration at soil sites for a wide range of earthquake magnitudes and distances with an error term, which is dependent on a number of additional factors (e.g.: event magnitude, event type and mechanism, rupture distance, site stiffness, etc.) A typical coefficient of variation term varies in the range of 30% to 40%. Any relevant information other than magnitude and distance should improve the accuracy of the estimations, which in turn should decrease the coefficient of variation (c.o.v.) to a value less than ∼ 0.35.

Similarly, comparisons of the actual recorded a_max_ values with site response analysis predictions based on “good” site characterization and seismic data revealed that the discrepancy in the matches is more typically in the range of c.o.v.10–20 %. The error represented by c.o.v. of a_max_ reduces to < 10% for the case history sites where actual strong ground motion recordings are available at the site of interest.

These outlined recommendations define guidelines for the estimation of mean and standard deviation for ln($a_{max}$). For illustration purposes, ln($a_{max}$)± $\sigma_{a_{max}}$ values are chosen as ln(0.28) ± 0.15.

#### Estimation of mean of moment magnitude

Due to use of different earthquake magnitude scales, a conversion factor may be needed to express the magnitude in moment magnitude scale. However, this conversion may not be exact. On the other hand, the reported magnitude might be in terms of moment magnitude but due to uncertainties in the estimations of the fault rupture dimensions, the rigidity of the ruptured material or for some other reason, the documented moment magnitude itself may not be exact. Due to the relative minor importance of these input parameters in the overall model, moment magnitude of the earthquake is incorporated as a deterministic value with mean of 6.8.

**Step 2: Estimation of Mean and Standard Deviations for Capacity and Demand Terms**

### Demand term: cyclic stress ratio, CSR

#### Seismic site response analyses

In-situ equivalent uniform ${CSR}_{{\sigma^{'}}_{v},\alpha ,M_{w}}$ can be evaluated either based on direct seismic site response analyses, or direct seismic site response and soil-structure-interaction analyses, as given by Eq. (25a,25b).

(25a) $$\tau_{av}\approx 0.65\cdot \tau_{max,site-response}$$

(25b) $${CSR}_{{\sigma^{\text{'}}}_{v},\alpha ,M_{w}}=\frac{\tau_{av}}{{\sigma \text{'}}_{v}}$$

#### Using the “Simplified” approach

##### Estimation of mean and standard deviation of nonlinear shear mass participation (Stress reduction) factor, $r_{d}$

The stress reduction or nonlinear shear mass participation factor, r_d_, is estimated as given by Cetin and Seed [22] in the form of Eqs. (26) and (27).

***For d< 20 m (∼65 ft):***

(26) $$r_{d}\left(d,M_{w},a_{max},V_{s,12}^{\text{*}}\right)=\frac{\left[1+\frac{-23.013-2.949\cdot a_{max}+0.999\cdot M_{w}+0.0525\cdot V_{s,12}^{\text{*}}}{16.258+0.201\cdot e^{0.341\cdot (-d+0.0785\cdot V_{s,12}^{\text{*}}+7.586)}}\right]}{\left[1+\frac{-23.013-2.949\cdot a_{max}+0.999\cdot M_{w}+0.0525\cdot V_{s,12}^{\text{*}}}{16.258+0.201\cdot e^{0.341\cdot (0.0785\cdot V_{s,12}^{\text{*}}+7.586)}}\right]}\pm \sigma_{\varepsilon_{r_{d}}}$$

***For d≥ 20 m (∼65 ft):***

(27) $$r_{d}\left(d,M_{w},a_{max},V_{s,12}^{\text{*}}\right)=\frac{\left[1+\frac{-23.013-2.949\cdot a_{max}+0.999\cdot M_{w}+0.0525\cdot V_{s,12}^{\text{*}}}{16.258+0.201\cdot e^{0.341\cdot (-20+0.0785\cdot V_{s,12}^{\text{*}}+7.586)}}\right]}{\left[1+\frac{-23.013-2.949\cdot a_{max}+0.999\cdot M_{w}+0.0525\cdot V_{s,12}^{\text{*}}}{16.258+0.201\cdot e^{0.341\cdot (0.0785\cdot V_{s,12}^{\text{*}}+7.586)}}\right]}-0.046\cdot (d-20)\pm \sigma_{\varepsilon_{r_{d}}}$$

The standard deviation of the model error term ($\sigma_{\varepsilon_{r_{d}}}$) is defined as given in Eq. (28):

**For d< 12 m (∼40 ft):**

(28a) $$\sigma_{\varepsilon_{r_{d}}}\left(d\right)=d^{0.850}\cdot 0.0198$$

**For d ≥ 12 m (∼40 ft):**

(28b) $$\sigma_{\varepsilon_{r_{d}}}\left(d\right)=12^{0.850}\cdot 0.0198$$

In Eqs. (26), (27), “d” is in meters and corresponds to the depth of interest (critical depth of 5.5 m for this case), a_max_ is in gravitational acceleration (in g’s), $V_{s,12m}^{*}$ is the time-averaged shear wave velocity over the top 12 m in m/sec calculated in the same manner as $V_{s,30}$, and “e” is the exponential symbol. A full explanation of the development of the probabilistic r_d_ relationship is presented in Cetin and Seed [22]. By inputting the corresponding values of the illustrative case, r_d_ and error term’s standard deviation are calculated as 0.97 and 0.084, respectively as presented in Eqs. (29) and (30).

(29) $$r_{d}\left(d,M_{w},a_{max},V_{s,12}^{\text{*}}\right)=\frac{\left[1+\frac{-23.013-2.949\cdot 0.28+0.999\cdot 6.8+0.0525\cdot 220}{16.258+0.201\cdot e^{0.341\cdot (-5.5+0.0785\cdot 220+7.586)}}\right]}{\left[1+\frac{-23.013-2.949\cdot 0.28+0.999\cdot 6.8+0.0525\cdot 220}{16.258+0.201\cdot e^{0.341\cdot (0.0785\cdot 220+7.586)}}\right]}=0.97$$

(30) $$\sigma_{\varepsilon_{r_{d}}}\left(5.5\right)=5.5^{0.850}\cdot 0.0198=0.084$$

##### Estimation of mean and standard deviation of $ln({CSR}_{{\sigma^{'}}_{v},\alpha ,M_{w}})$ by simplified procedure

In-situ equivalent uniform ${CSR}_{{\sigma^{'}}_{v},\alpha ,M_{w}}$ can be evaluated based on the "simplified" approach by employing Eq. (31) along with the $r_{d}$ relationships given by Cetin and Seed [22] as given in Eq. (29).

(31) $${CSR}_{{\sigma \text{'}}_{v},M_{w}}=0.65\cdot {CSR}_{peak,{\sigma \text{'}}_{v},M_{w}}=0.65\cdot \frac{a_{max}}{g}\cdot \frac{\sigma_{v}}{{\sigma \text{'}}_{v}}\cdot r_{d}$$

The mean ${CSR}_{{\sigma^{'}}_{v},\alpha ,M_{w}}$ for the critical layer of illustrative case can be estimated as 0.23 as presented in Eq. (32).

(32) $$\mu_{{CSR}_{{\sigma \text{'}}_{v},M_{w}}}≅\frac{0.65\cdot \mu_{a_{max}}\cdot \mu_{\sigma_{v}}\cdot \mu_{r_{d}}}{g\cdot \mu_{{\sigma \text{'}}_{v}}}=\frac{0.65\cdot 0.28g\cdot 105.2\cdot 0.97}{g\cdot 80.7}=0.23$$

Similarly, the uncertainty in CSR, where only the total stress and effective stress terms are assumed to be correlated, can be estimated as given in Eq. (33).

(33) $$\delta_{CSR}^{2}=\delta_{a_{max}}^{2}+\delta_{r_{d}}^{2}+\delta_{\sigma_{v}}^{2}+\delta_{{\sigma \text{'}}_{v}}^{2}-2\cdot \delta_{\sigma_{v}}\cdot \delta_{{\sigma \text{'}}_{v}}\cdot \rho_{\sigma_{v}\cdot {\sigma \text{'}}_{v}}$$

Coefficient of variation (δ) of each term can be calculated by the ratio of standard deviation to mean (σ/μ). Thus, Eq. (33) can be re-written as given in Eq. (34); whereas, the correlation coefficient between total and effective stress terms is defined as given in Eq. (35).

(34) $$\delta_{CSR}^{2}={\left(\frac{\sigma_{CSR}}{\mu_{CSR}}\right)}^{2}={\left(\frac{\sigma_{a_{max}}}{\mu_{a_{max}}}\right)}^{2}+{\left(\frac{\sigma_{r_{d}}}{\mu_{r_{d}}}\right)}^{2}+{\left(\frac{\sigma_{\sigma_{v}}}{\mu_{\sigma_{v}}}\right)}^{2}+{\left(\frac{\sigma_{{\sigma \text{'}}_{v}}}{\mu_{{\sigma \text{'}}_{v}}}\right)}^{2}-2\cdot \left(\frac{\sigma_{\sigma_{v}}}{\mu_{\sigma_{v}}}\right)\cdot \left(\frac{\sigma_{{\sigma \text{'}}_{v}}}{\mu_{{\sigma \text{'}}_{v}}}\right)\cdot \rho_{\sigma_{v}\cdot {\sigma \text{'}}_{v}}$$

(35) $$\rho_{\sigma_{v}\cdot {\sigma \text{'}}_{v}}=\frac{h_{w}^{2}\cdot \sigma_{\gamma_{1}}^{2}+\left(\gamma_{1}-\gamma_{2}\right)\cdot \left(\gamma_{1}+\gamma_{w}-\gamma_{2}\right)\cdot \sigma_{h_{w}}^{2}+{\left(h-h_{w}\right)}^{2}\cdot \sigma_{\gamma_{2}}^{2}+\gamma_{2}\cdot \left(\gamma_{2}-\gamma_{w}\right)\cdot \sigma_{h}^{2}}{\sigma_{\sigma_{v}}\cdot \sigma_{{\sigma \text{'}}_{v}}}$$

By substituting the corresponding values into these equations, $\rho_{\sigma_{v}\cdot {\sigma '}_{v}}$, $\delta_{CSR}^{2}$ and $\sigma_{CSR}$ can be determined as given in Eqs. (36), (37), (38), respectively.

(36) $$\rho_{\sigma_{v}\cdot {\sigma \text{'}}_{v}}=\frac{3^{2}\cdot 0.5^{2}+\left(18.4-20\right)\cdot \left(18.4+9.81-20\right)\cdot 1^{2}+{\left(5.5-3\right)}^{2}\cdot 0.5^{2}+20\cdot \left(20-9.81\right)\cdot 0.83^{2}}{16.8\cdot 12}=0.65$$

(37) $$\delta_{CSR}^{2}={\left(\frac{0.15}{0.28}\right)}^{2}+{\left(\frac{0.084}{0.97}\right)}^{2}+{\left(\frac{16.8}{105.2}\right)}^{2}+{\left(\frac{12}{80.7}\right)}^{2}-2\cdot \left(\frac{16.8}{105.2}\right)\cdot \left(\frac{12}{80.7}\right)\cdot 0.65=0.31$$

(38) $$\sigma_{CSR}=\sqrt{\delta_{CSR}^{2}\cdot \mu_{CSR}^{2}}=\sqrt{0.31\cdot 0.23^{2}}=0.128$$

### Capacity term: cyclic resistance ratio, ${CRR}_{{\sigma \text{'}}_{v},\alpha ,M_{w}}$ or ${CRR}_{{\sigma \text{'}}_{v}=100\;kPa,\alpha =0,M_{w}=7.5}$

#### Determination of ${CRR}_{{\sigma '}_{v},\alpha ,M_{w}}$ by closed form Eq. (2)

CRR term corresponding to any vertical effective stress ratio and moment magnitude can be estimated by using the equation given in Eq. (2). As recommended by CEA2018, this equation will be solved for probability of liquefaction of 50%,for which ${\text{Φ}}^{-1}\left(P_{L}=50\%\right)$ term is equal to zero. Hence, as presented in Eq. (39), CRR is calculated as 0.15.

(39) CRRN1,60,Mw,σ'v,FC,PL=exp11.4∙1+0.00167⋅14-27.352⋅ln6.8-3.958⋅ln80.7101.3+0.089⋅14+16.084+2.95⋅0.011.771=0.15

#### Determination of ${CRR}_{{\sigma \text{'}}_{v}=100\;kPa,\alpha =0,M_{w}=7.5}$ from $N_{1,60,CS}$ vs. CRR figure

The proposed methodology also allows the estimation of CRR corresponding to reference stress (i.e. ${\sigma^{'}}_{v}$ = 100 kPa and α = 0.0) and moment magnitude ($M_{w}$ = 7.5) states by using the chart solution given in Fig. 1. However, series of corrections need to be applied to convert this reference CRR value to the site and event specific CRR value. These corrections will be discussed later in the text.

##### Determination of $N_{1,60,CS}$

**Fig. 4:**
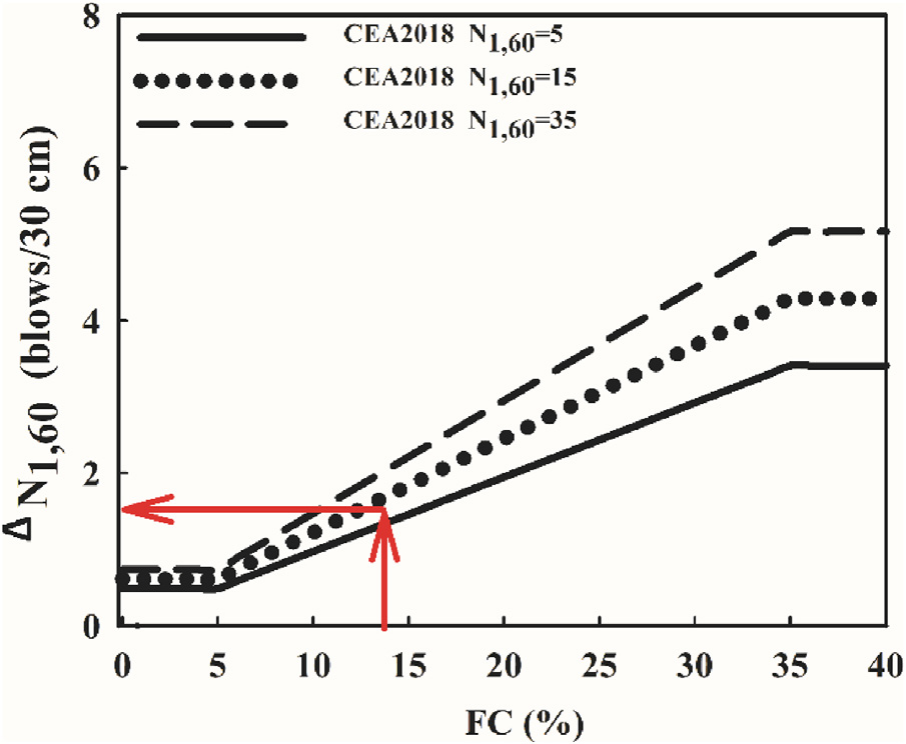
Proposed N_1,60_ dependent fines correction.

$N_{1,60}$ -values calculated in Step 1.A.1 must then be further corrected for fines effects to determine $N_{1,60,CS}$ -values, by using either Eqs. (40) and (41) or Fig. 4. Fig. 4 presents the regressed fines corrections of CEA2018.

(40) $$N_{1,60,CS}=N_{1,60}+\text{Δ}N_{1,60}$$

(41) $$\text{Δ}N_{1,60}=FC\cdot (\theta_{1}\cdot N_{1,60}+\theta_{4})$$

lim: 5% ≤ FC ≤ 35%

By substituting the mean $N_{1,60}$ and FC values along with the $\theta_{i}$ values given in Table 1, fines corrected SPT-N value can be determined as 13 blows/ft., as presented in Eq. (42).

(42) $$N_{1,60,CS}=11.4+14\cdot \left(0.00167\cdot 11.4+0.089\right)=12.9≅13$$

##### Determination of ${CRR}_{{\sigma \text{'}}_{v}=100\;kPa,\alpha =0,M_{w}=7.5}$

**Fig. 5:**
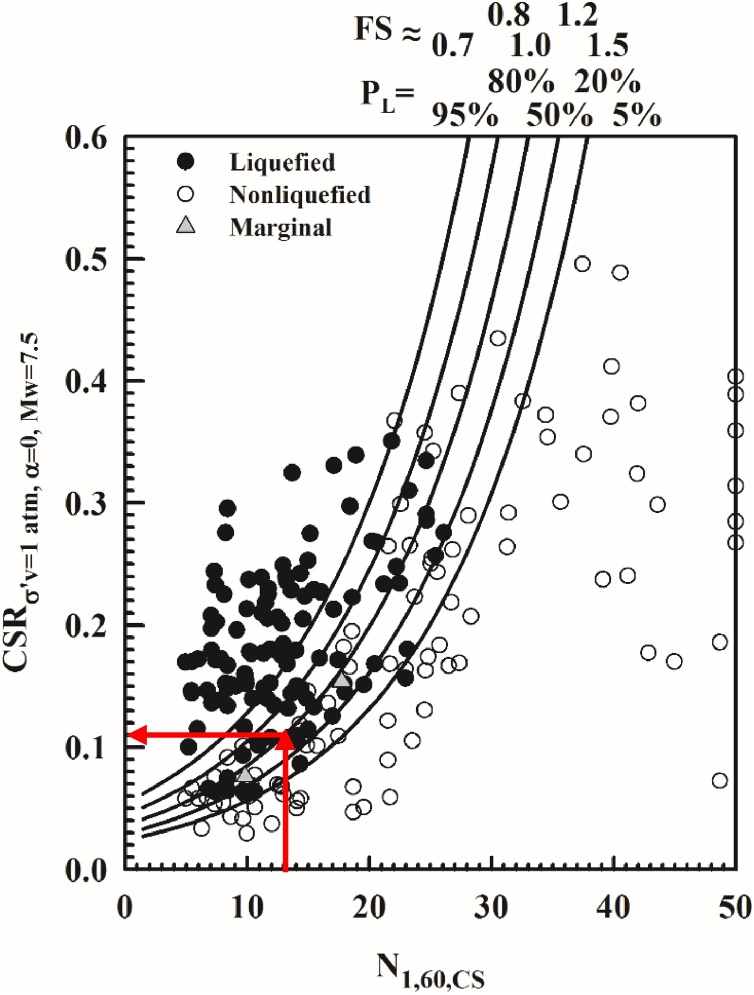
Use of new probabilistic seismic soil liquefaction triggering curves for estimation CRR.

${CRR}_{{\sigma '}_{v}=100\;kPa,\alpha =0,M_{w}=7.5}$ can be estimated by using the proposed probabilistic boundary curves,which were developed by considering the uncertainty due to model error only, as presented in Fig. 5. Consistent with Step 2.B.2.1, $P_{L}$ = 50% curve is used and then for input $N_{1,60,CS}$ value of 13 blows/ft, corresponding ${CRR}_{{\sigma '}_{v}=100\;kPa,\alpha =0,M_{w}=7.5}$ value is determined as 0.11.

**Step 3: Seismic Soil Liquefaction Triggering Performance Assessments**

#### Determination of probability of liquefaction triggering, $P_{L}$

##### Determination of P_L_ by the closed form Eq. (1)

Probability of liquefaction can be estimated by using Eq. (1) as 96% as presented in Eq. (43).

(43) $$P_{L}\left(N_{1,60},CSR_{{\sigma^{\text{'}}}_{v},\alpha =0,M_{w}},M_{w},{\sigma^{\text{'}}}_{v},FC\right)=\text{Φ}\left[-\frac{\left(\begin{array}{c} 11.4∙\left(1+0.00167\cdot 14\right)-11.771\cdot \text{ln}\left(0.23\right)\\ -27.352\cdot \text{ln}\left(6.8\right)-3.958\cdot ln\left(\frac{80.7}{101.3}\right)+0.089\cdot 14+16.084 \end{array}\right)}{2.95}\right]=\text{Φ}\left[1.77\right]=0.96$$

Φ is the standard cumulative normal distribution. For spreadsheet construction purposes, the command in Microsoft Excel for this specific function is “NORMDIST(P_L_;0;1;TRUE)”.

#### Determination of $P_{L}$ from $N_{1,60,CS}$ vs. CRR curves given in Fig. 1

Use of chart solution requires series of corrections which will be introduced in following sections.

##### Determination of $N_{1,60,CS}$

As discussed in Step 2.B.2.1, $N_{1,60,CS}$ is 13 blows / ft.

##### Determination of ${CSR}_{{\sigma^{\text{'}}}_{v},\alpha ,M_{w}}$

As discussed in Step 2.A.2.2, ${CSR}_{{\sigma '}_{v},M_{w}}$ is 0.23.

##### Estimation of $K_{\sigma }$

**Fig. 6:**
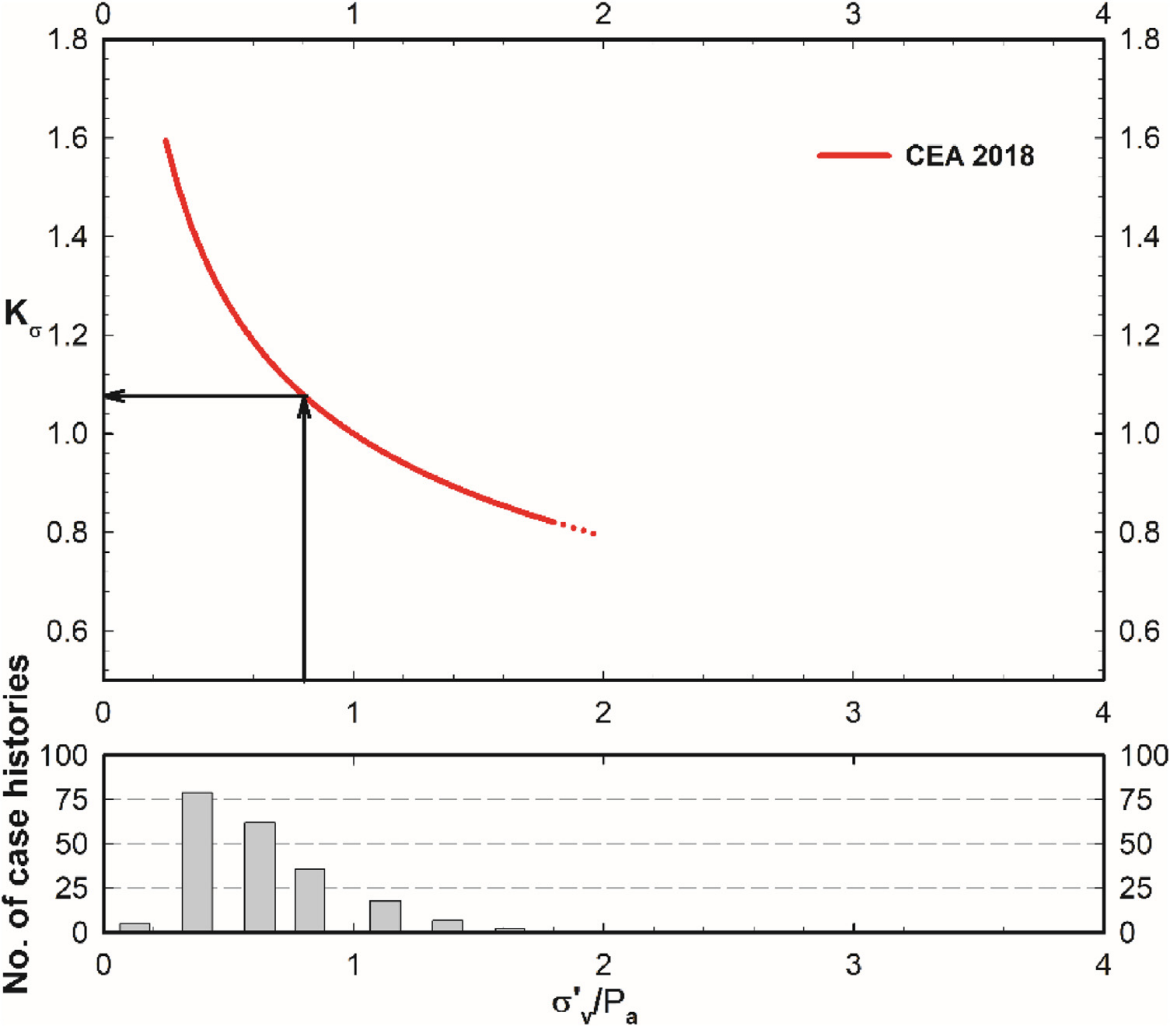
Use of proposed $K_{\sigma }$ curve for the illustrative case.

For the estimation of stress-scaling factor, $K_{\sigma }$, either the closed form solution given in Eq. (44) or the chart solution presented in Fig. 6 can be used. This relationship was developed based on the regression of the liquefaction performance field case history database. The histogram of the vertical effective stresses of these case histories is also presented in Fig. 6. Hence the use of the proposed $K_{\sigma }$ relationship should be limited over the effective vertical stress range of 0.25 atm. ≤ σ'_v_ ≤ 1.8 atm. Extrapolation to higher vertical effective stresses beyond 1.8 atm. for forward engineering analyses is controversial, and the readers are referred to the discussion presented in Cetin et al. [26].

(44) $$K_{\sigma }={\left(\frac{{\sigma \text{'}}_{v}}{P_{a}}\right)}^{-\theta_{3}/\theta_{6}}={\left(\frac{{\sigma \text{'}}_{v}}{P_{a}}\right)}^{-3.958/11.771}={\left(\frac{{\sigma \text{'}}_{v}}{P_{a}}\right)}^{-0.336}$$

lim: 0.25 atm ≤ ${\sigma '}_{v}$ ≤ 1.8 atm

Using the corresponding ${\sigma '}_{v}$ of the critical layer, $K_{\sigma }$ can be determined as 1.08 as presented in Eq. (45) and Fig. 6.

(45) $$K_{\sigma }={\left(\frac{80.7}{101.3}\right)}^{-0.336}=1.08$$

##### Estimation of $K_{M_{w}}$

**Fig. 7:**
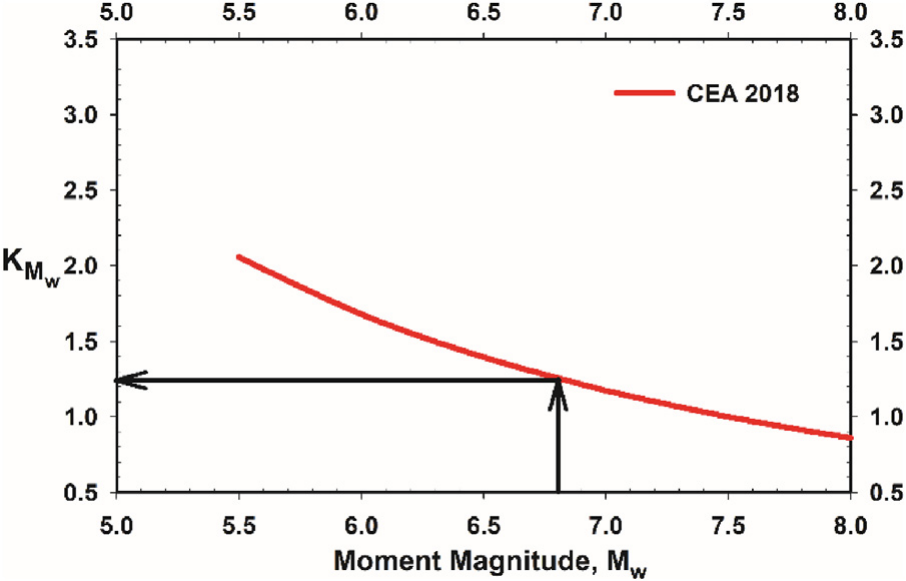
Use of Proposed $K_{M_{w}}$ curves for the illustrative case.

For the estimation of seismic moment magnitude (duration) scaling factor, $K_{M_{w}}$, either the closed form solution given in Eq. (46) or the chart solution presented in Fig. 7 can be used. This relationship was developed based on the regression of the liquefaction performance field case history database.

(46) $$K_{M_{w}}={\left(\frac{M_{w}}{7.5}\right)}^{-\theta_{2}/\theta_{6}}={\left(\frac{M_{w}}{7.5}\right)}^{-27.352/11.771}={\left(\frac{M_{w}}{7.5}\right)}^{-2.324}$$

For the $M_{w}=6.8$ seismic event, $K_{M_{w}}$ can be determined as 1.26 as presented in Eq. (47) and Fig. 7.

(47) $$K_{M_{w}}={\left(\frac{6.8}{7.5}\right)}^{-2.324}=1.26$$

##### Estimation of $K_{\alpha }$

As the illustrative soil site is free field level site, there exists no shear stresses acting on the horizontal plane (i.e.: α = 0**)**. Hence no correction for $K_{\alpha }$ effects is needed (i.e.: $K_{\alpha }$ = 1.0**)**. However, it should be noted that at sloping soil sites, and at soil sites where a super-structure is overlying potentially liquefiable soils, $K_{\alpha }$ correction needs to be applied as recommended in Harder and Boulanger [27], Boulanger [28], Cetin and Bilge [29].

##### Estimation of ${CSR}_{{\sigma \text{'}}_{v}=100\;kPa,\alpha =0,M_{w}=7.5}$

${CSR}_{{\sigma '}_{v},\alpha ,M_{w}}$ is then adjusted with $K_{\sigma }$, $K_{\alpha }$ and $K_{M_{w}}$ correction factors to convert the field and event specific CSR value to the reference CSR value, valid for ${\sigma '}_{v}$ = 1 atm, α = 0 and $M_{w}$ = 7.5, as given in Eq. (48). Note that for level sites, $K_{\alpha }$ = 1.0.

(48) $${CSR}_{{\sigma \text{'}}_{v}=1atm,\alpha =0,M_{w}=7.5}={CSR}_{{\sigma \text{'}}_{v},\alpha ,M_{w}}\cdot \frac{1}{K_{\sigma }}\cdot \frac{1}{K_{M_{w}}}\cdot \frac{1}{K_{\alpha }}$$

lim: ${CSR}_{{\sigma '}_{v}=1atm,\alpha =0,M_{w}=7.5}$ ≤0.6

The mean value of ${CSR}_{{\sigma '}_{v}=1atm,\alpha =0,M_{w}=7.5}$ for the critical layer is estimated as 0.17 as given in Eq. (49).

(49) $${CSR}_{{\sigma \text{'}}_{v}=1atm,\alpha =0,M_{w}=7.5}=0.23\cdot \frac{1}{1.08}\cdot \frac{1}{1.26}\cdot \frac{1}{1.0}=0.17$$

##### Estimation of probability of liquefaction, $P_{L}$

**Fig. 8:**
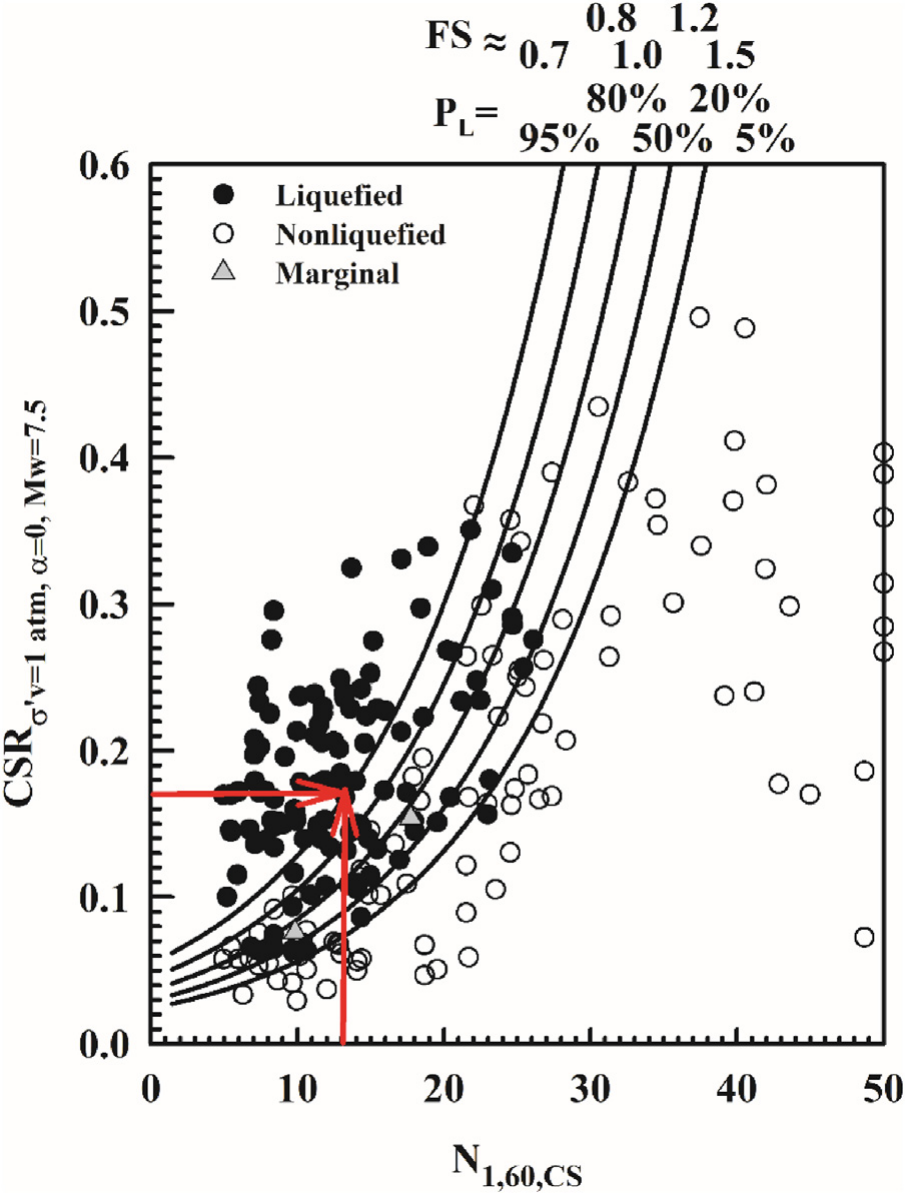
Use of new probabilistic seismic soil liquefaction triggering curves for estimation of P_L._

The resulting, fully adjusted and normalized values of $N_{1,60,CS}$ and ${CSR}_{{\sigma '}_{v}=1atm,\alpha =0,M_{w}=7.5}$ can then be used as shown in Fig. 8 to estimate the probability of liquefaction triggering as 96%. It should be noted that these curves were developed by considering the uncertainty due to model error only. If the uncertainty of input parameters needs to be incorporated into the assessment, then Fig. 1 cannot be used. For $N_{1,60,CS}$ = 13 blows / ft and ${CSR}_{{\sigma '}_{v}=1atm,\alpha =0,M_{w}=7.5}=0.17\;$, $P_{L}$ is estimated from Fig. 8 as 96%.

As discussed earlier, Eqs. (1) and (2) are only applicable for cases where the input parameters are exact. However, usually there exists significant uncertainty associated with these parameters in typical design applications. The proposed framework of CEA2018 allows assessment of the parameter uncertainty in forward engineering problems. For the cases where input parameters are uncertain, then in Eq. (1), instead of model error ($\sigma_{\varepsilon }$), the overall standard deviation term ($\sigma_{tot}$) needs to be used along with Eqs. (4) and (5). By substituting the corresponding mean input parameters, the overall (consolidated) uncertainty of the illustrative case ($\sigma_{input}$) can be calculated as presented in Eq. (50).

(50) $$\sigma_{input}^{2}=11.771^{2}\cdot 0.31+2.3^{2}\cdot {\left(1+0.00167\cdot 14\right)}^{2}+5.3^{2}\cdot {\left(0.00167\cdot 14+0.089\right)}^{2}+3.958^{2}\cdot {\left[\frac{12}{80.7}\right]}^{2}=49.2\;\;\;\sigma_{input}=7.01$$

By inputting $\sigma_{input}$ along with related model parameters of CEA2018 into Eq. (4), the overall uncertainty for the illustrative can be calculated as given in Eq. (51).

(51a) $$\sigma_{tot}^{2}={\left(0.392\cdot 7.01\right)}^{2}+2.95^{2}=16.25$$

(51b) $$\sigma_{tot}=4.03$$

When the uncertainties of the input parameters are considered, then the probability of liquefaction triggering is estimated as 90% as presented in Eq. (51c).

(51c) $$P_{L}\left(N_{1,60},CSR_{{\sigma^{\text{'}}}_{v},\alpha =0,M_{w}},M_{w},{\sigma^{\text{'}}}_{v},FC\right)=\text{Φ}\left[-\frac{\left(\begin{array}{c} 11.4∙\left(1+0.00167\cdot 14\right)-11.771\cdot \text{ln}\left(0.23\right)\\ -27.352\cdot \text{ln}\left(6.8\right)-3.958\cdot ln\left(\frac{80.7}{101.3}\right)+0.089\cdot 14+16.084 \end{array}\right)}{4.03}\right]=\text{Φ}\left[1.30\right]=0.90$$

### Determination of factor of safety against liquefaction triggering, FS

For the “deterministic” evaluation of liquefaction triggering, Eq. (52), which defines the ratio of capacity to demand terms, can be used to estimate the factor safety against liquefaction triggering.

(52) $$FS=\frac{CRR}{CSR}=\frac{{CRR}_{{\sigma \text{'}}_{v},\alpha ,M_{w}}}{{CSR}_{{\sigma \text{'}}_{v},\alpha ,M_{w}}}=\frac{{CRR}_{{\sigma \text{'}}_{v}=1atm,\alpha =0,M_{w}=7.5}}{{CSR}_{{\sigma \text{'}}_{v}=1atm,\alpha =0,M_{w}=7.5}}$$

While using Eq. (52), it is vital to adjust the CSR and CRR terms to same stress and magnitude (duration) states. Thus, FS can be calculated by either using the values corresponding to the site and event specific state or adjusted to the reference state (i.e. ${\sigma '}_{v}=1atm,\alpha =0,M_{w}=7.5$) as given by Eqs. (53) and (54), respectively.

(53) $$FS=\frac{{CRR}_{{\sigma \text{'}}_{v},\alpha ,M_{w}}}{{CSR}_{{\sigma \text{'}}_{v},\alpha ,M_{w}}}=\frac{0.15}{0.23}=0.65$$

(54) $$FS=\frac{{CRR}_{{\sigma \text{'}}_{v}=1atm,\alpha =0,M_{w}=7.5}}{{CSR}_{{\sigma \text{'}}_{v}=1atm,\alpha =0,M_{w}=7.5}}=\frac{0.11}{0.17}=0.65$$
